# Reanalysis of a μ opioid receptor crystal structure reveals a covalent adduct with BU72

**DOI:** 10.1186/s12915-023-01689-w

**Published:** 2023-10-10

**Authors:** Thomas A. Munro

**Affiliations:** School of Life and Environmental Sciences, Deakin University, Burwood, VIC 3125 Australia

**Keywords:** BU72, Covalent adduct, Crystal structure, μ opioid receptor, Revised stereochemistry

## Abstract

**Background:**

The first crystal structure of the active μ opioid receptor (μOR) exhibited several unexplained features. The ligand BU72 exhibited many extreme deviations from ideal geometry, along with unexplained electron density. I previously showed that inverting the benzylic configuration resolved these problems, establishing revised stereochemistry of BU72 and its analog BU74. However, another problem remains unresolved: additional unexplained electron density contacts both BU72 and a histidine residue in the N-terminus, revealing the presence of an as-yet unidentified atom.

**Results:**

These short contacts and uninterrupted density are inconsistent with non-covalent interactions. Therefore, BU72 and μOR form a covalent adduct, rather than representing two separate entities as in the original model. A subsequently proposed magnesium complex is inconsistent with multiple lines of evidence. However, oxygen fits the unexplained density well. While the structure I propose is tentative, similar adducts have been reported previously in the presence of reactive oxygen species. Moreover, known sources of reactive oxygen species were present: HEPES buffer, nickel ions, and a sequence motif that forms redox-active nickel complexes. This motif contacts the unexplained density. The adduct exhibits severe strain, and the tethered N-terminus forms contacts with adjacent residues. These forces, along with the nanobody used as a G protein substitute, would be expected to influence the receptor conformation. Consistent with this, the intracellular end of the structure differs markedly from subsequent structures of active μOR bound to G_i_ protein.

**Conclusions:**

Later G_i_-bound structures are likely to be more accurate templates for ligand docking and modelling of active G protein-bound μOR. The possibility of reactions like this should be considered in the choice of protein truncation sites and purification conditions, and in the interpretation of excess or unexplained density.

**Supplementary Information:**

The online version contains supplementary material available at 10.1186/s12915-023-01689-w.

## Background

**Fig. 1:**
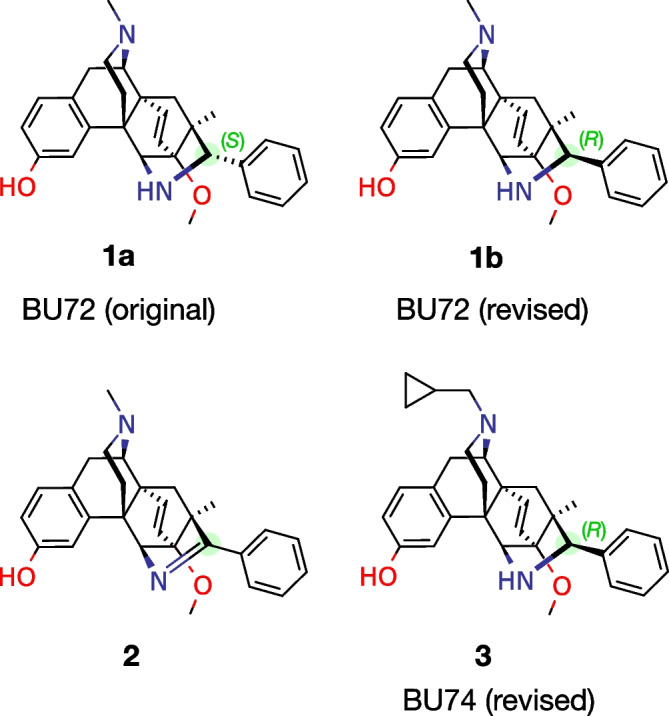
Structures of BU72 and analogs

BU72 is a μ opioid of exceptionally high binding affinity and potency (Fig. 1) [1, 2]. Its dissociation constant (*K*_i_) for μOR ranges from 0.15 nM in crude brain membranes [1], to lower values in transfected cell membranes [2, 3], and as low as 0.01 nM for purified μOR with G_i_ protein [3]. Very few ligands for any protein exceed this extraordinary affinity, which is considered an effective upper bound on the strength of non-covalent binding [4].

BU72 was the ligand used in the first crystal structure of active μOR [3]. As noted there, the structure exhibited two unexplained features. Firstly, fitting BU72 (**1a**, Fig. 1) required an implausibly high-energy conformation, with many extreme deviations from ideal geometry. The authors considered the possibility that the ligand was actually imine **2**, but this was not detected in the crystallization mixture [3]. I later proposed an alternative: a revised structure for BU72 with the phenyl group in the opposite (*R*) configuration **1b**; this permitted a more plausible low-energy conformation and a better fit to the density [5]. The authors of the crystal structure, including the lead author of the original synthesis of BU72, accepted this revision [6]. Note that the structure of the analog BU74 (**3**) should also be revised, since they differ only in the N-substituent [7]; the synthetic routes diverge after establishment of the phenyl configuration, and the benzylic hydrogen is not exchangeable.

**Fig. 2:**
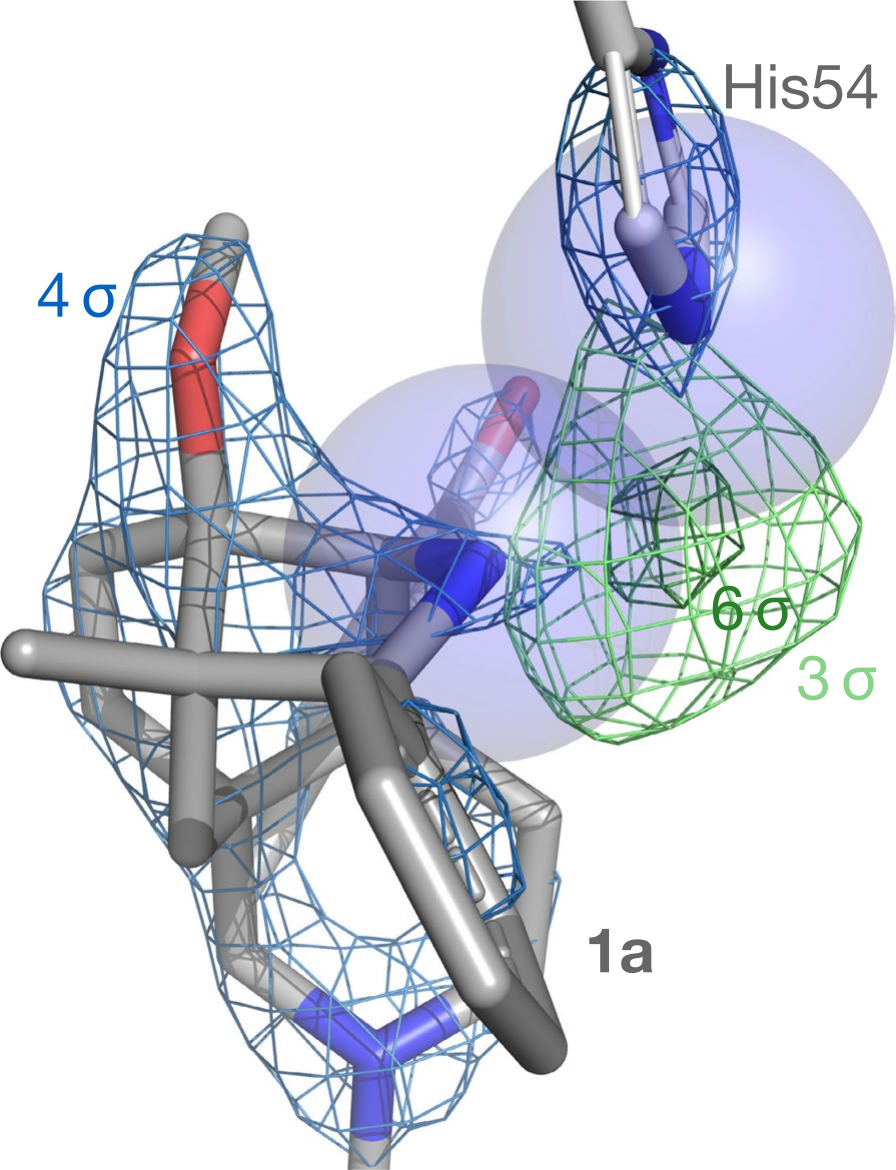
Clashes and unexplained density between BU72 and His54 in the original model (PDB 5C1M v.1.5 [8]). *2F*o-*F*c density (*blue*) and *F*o-*F*c omit density (*green*) are shown at the indicated levels. Clashing N atoms are shown as spheres

However, a second puzzling feature of the crystal structure remains unexplained after this revision. The truncated N-terminus of the receptor, which is unresolved in other opioid receptor structures due to disorder, unexpectedly forms a “lid” over the binding pocket [3]. The third residue of the terminus, His54, clashes with BU72. The overlapping atoms also contact a pocket of strong, unexplained electron density (Fig. 2). The atom responsible for this density was not identified: experiments testing for an alternative ligand or a coordinated heavy metal ion were unsuccessful [3]. The atom was ultimately omitted from the model altogether.

Other authors later proposed that the missing atom is a magnesium ion; this fitted the unexplained density well, while lithium, sodium, nickel, and zinc ions did not [9]. Bond lengths were not given, but were reportedly consistent with a magnesium coordination complex [10]. Below I evaluate this proposal and consider alternatives.

## Results

### The missing atom is not magnesium

**Fig. 3:**
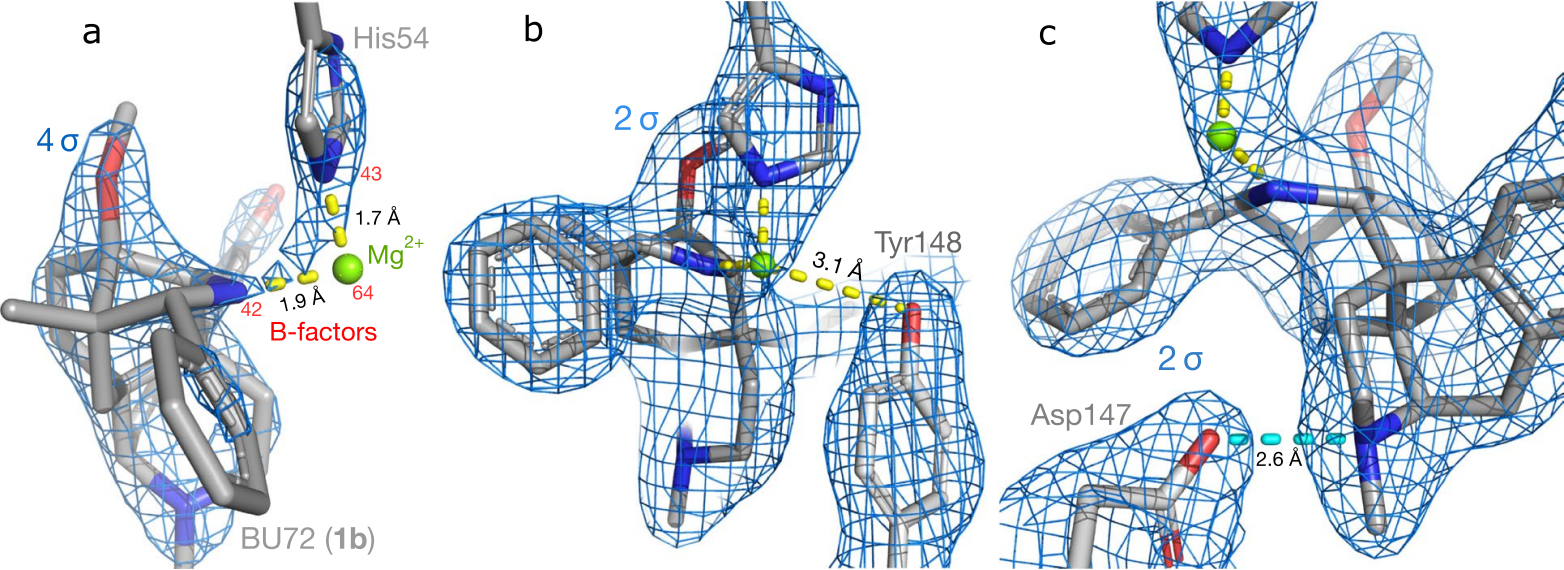
Proposed magnesium complex. **a** Bond lengths and B-factors (*red*). **b** Proposed third bond from Mg^2+^ to Tyr148^3x33^. **c** Comparison with the salt bridge to Asp147^3x32^

Placing an Mg^2+^ ion in the unexplained density followed by refinement confirmed the earlier reports of a good fit, with no excess or unexplained density above 2.5 σ (Fig. 3a; data in Additional files 1 and 2). However, contrary to these prior reports, the N–Mg bonds were unrealistically short (1.9 and 1.7 Å). Compare the N–Mg bond lengths in structures of subatomic resolution: 2.19 ± 0.06 Å, mean ± s.d. (standard deviation) [11]. These proposed bonds are thus extreme outliers, with *Z* scores of −5 and −9, respectively. The high resolution of the structure (2.1 Å) allows strong conclusions about bond lengths, with a diffraction precision index (DPI) of 0.22 Å for the Mg^2+^ ion [12]. Note also that despite these short distances, the ion was not centered in the density, suggesting that the actual bonds must be even shorter (Fig. 3a). This resulted in a poor real-space R value (RSR) of 0.32 for the Mg^2+^ ion, despite good values for His54 (0.11) and BU72 (0.08).

A later report from the same group added a third bond to the model [10], from Mg^2+^ to Tyr148^3x33^ (Fig. 3b), expressed in generic GPCRdb numbering [13]. However, this proposal requires an O−Mg bond length of 3.1 Å; compared with high-resolution structures (2.10 ± 0.04 Å), this is untenable (*Z* = 25) [11]. It is instead suggestive of a hydrogen bond to another element. Note also the large gap in the electron density along this proposed bond, unlike the strong and uninterrupted density for the other bonds (Fig. 3b). Additionally, note the highly asymmetrical geometry required, with a bond angle of 105°, compared to 90° for the N atoms: magnesium complexes are symmetrical [11].

Other evidence against Mg^2+^ was revealed by CheckMyMetal, a metal binding site validation server [14]. The values of five of the eight parameters evaluated were classed as dubious, including three that strongly suggest a misidentified element:

- A much higher temperature factor (B-factor) for the ion than the bonding partners (Fig. 3a); since bonds transmit thermal motion, this is implausible [15].
- Bonding to a protonated amine (NH^+^); Mg^2+^ favors neutral or negatively charged bonding partners [16].
- An incomplete coordination sphere. The expected number of bonds is six, or in rare cases four or five; a value of two is extremely rare in high-resolution structures [17].

While unresolved water molecules might complete the coordination sphere, this is implausible since the rest of the complex is well resolved with full occupancy, as are many structured water molecules elsewhere in the binding pocket [3].

Finally, no source of magnesium is mentioned in the experimental method [3]. Collectively, the above evidence firmly excludes Mg^2+^ as a candidate.

### The missing atom forms covalent bonds to both BU72 and His54

The fit of the Mg^2+^ ion to the density establishes that a non-hydrogen atom is present in this approximate position. As noted above, this missing atom is likely nearer to both His54 and BU72 than the modelled position of Mg^2+^; that is, < 1.9 Å from each (Fig. 3a). This is much too close for non-covalent interactions (≥ 2.4 Å) [18], which would also not result in strong, uninterrupted electron density connecting the three atoms. For instance, the protonated tertiary amine of BU72 forms a charge-assisted hydrogen bond to Asp147^3x32^ (Fig. 3c); these are among the shortest of all noncovalent interactions [18]. Nonetheless, the N⋯O distance is 2.6 Å, and the regions of high electron density are well separated, in striking contrast to the continuous density surrounding the proposed Mg^2+^ complex. Therefore, the unidentified atom is covalently bonded to both BU72 and μOR; that is, they form an adduct.

While this evidence does not definitively establish the identity of the missing atom, it is inconsistent with the published model of BU72 and the receptor as discrete entities. One way to resolve this would be to model the adduct, but leave the bridging atom unidentified. Many Protein Data Bank (PDB) models include unidentified atoms (ligand identifier UNX). Nonetheless, the evidence is sufficient to exclude some elements, as discussed below.

### The missing atom is very unlikely to be a metal, but may be oxygen

The CheckMyMetal validation report for the magnesium complex suggested alternative metals as better candidates: copper, iron, cobalt, nickel, manganese, and zinc. However, each of these also gave multiple outliers when validated. Also, of these metals, only nickel was present during preparation of the crystals; it was used for affinity purification [3]. The bond lengths are more plausible than for magnesium, since N−Ni bonds are short (1.88 ± 0.03 Å) [11]. However, as noted above, nickel did not fit the electron density, leaving a substantial excess [9]. Further evidence against nickel and other heavy metals is the lack of anomalous scattering noted in the original report [3].

The only metal in the buffer solution, sodium, also gave five dubious values in CheckMyMetal, including even more extreme outliers from typical N−Na bond lengths (2.46 ± 0.02 Å, Z =  −29 and −40) [11], and a much worse fit to the density than magnesium [9]. Indeed, no metal forms coordination bonds to N shorter than 1.76 Å [11]. It is thus extremely implausible that the missing atom is a metal.

Given the above, it appears that the missing atom is a non-metal approximately isoelectronic with magnesium, but forming shorter bonds. The element must also be at least divalent, and can probably form hydrogen bonds given its distance to Tyr148^3x33^ (~ 3.1 Å). One candidate meeting these criteria is oxygen; water molecules in crystal structures are frequently misidentified as magnesium [19].

### A known source of reactive oxygen species contacts the unexplained density

Formation of an oxygen-bridged adduct between the secondary amine of BU72 and the imidazole ring of His54 would require harsh conditions. Reactive oxygen species (ROS), for instance, can oxidize secondary amines [20] and histidine [21]. But how might these ROS arise? Surprisingly, several potential sources were present. The BU72-μOR complex was purified and crystallized in HEPES buffer, which generates hydrogen peroxide on exposure to light [22]. HEPES has also been reported to enhance metal-catalyzed generation of other ROS from hydrogen peroxide [23]. A further potential source is the N-terminus, which contains a sequence motif known to generate ROS. The N-terminus used was truncated, leaving glycine as the first residue and histidine as the third [3]. This sequence motif (H-Gly-Xaa-His-) forms redox-active nickel coordination complexes [24]. Moreover, a nickel affinity column was used for purification [3], and the H-Gly-Xaa-His- motif can capture Ni^2+^ ions from these columns [25–27]. The resulting square planar nickel complexes catalyze the decomposition of hydrogen peroxide to other ROS, including the hydroxyl radical [24, 28], which has been described as “the most reactive biological oxidant” [29]. Thus, the conditions used were sufficient to generate ROS near His54, potentially oxidizing both the residue itself and BU72.

A search of PDBeMotif [30] revealed eight protein structures in which square planar Ni^2+^-Gly-Xaa-His- complexes were resolved: PDB entries 1JVN [31], 1XMK [32], 2RJ2 [33], 3RDH [34], 3UM9 [35], 3ZUC [36], 4I71 [37], and 4OMO [38]. In three of these cases, the nickel was not added during crystallization, but unexpectedly captured during affinity chromatography: 1JVN [25], 3UM9 [26], and 3ZUC [27]. Intriguingly, in 1JVN the electron density was not consistent with the expected ligand structure; no density supported several of the atoms, suggesting partial decomposition [25]. The buffer used, PIPES, is an analog of HEPES that also generates hydrogen peroxide [39] and other ROS [23]. This provides a plausible explanation for the decomposition of the ligand.

### Proposed formation and structure of an oxygen-bridged adduct

**Fig. 4:**
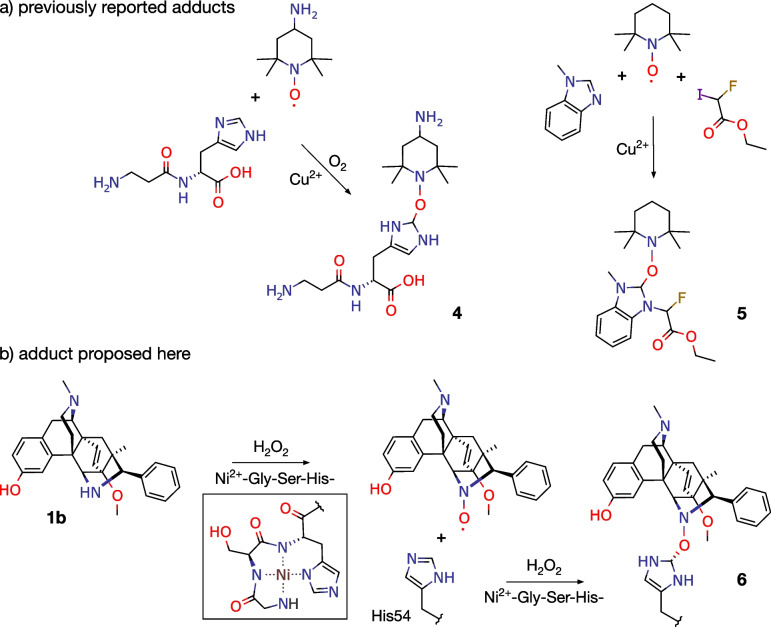
Adduct structures. **a** Previously reported adducts **4** ([21], Fig. 7c) and **5** ([40], Scheme 2). **b** Proposed adduct **6**, with the nickel complex and a possible aminoxyl intermediate

Two previous reports of adduct formation between aminoxyl radicals and imidazole rings are shown in Fig. 4a [21, 40]. These suggested potential structure **6** for an adduct between BU72 and His54 (Fig. 4b). The stereochemistry of the histidine derivative was dictated by the observed density. A possible intermediate aminoxyl radical is also shown; these can form via oxidation of secondary amines by ROS [20].

This proposal finds support in a puzzling result from the original report. Despite the very strong interactions apparent between BU72 and His54, removal of the side-chain of His54 by receptor mutagenesis had no detectable effect on the affinity or potency of BU72 [3]. This seeming paradox, however, is consistent with the mechanism proposed here. Affinity and potency were measured using cells and cell membranes rather than purified proteins, so no nickel was added. Moreover, the cells expressed the full-length receptor, which lacks the N-terminal motif that forms nickel complexes [24]. Thus, the reactions proposed above could not occur, and the assays would be unaffected by the presence or absence of His54.

### The oxygen-bridged adduct fits the unexplained density

**Fig. 5:**
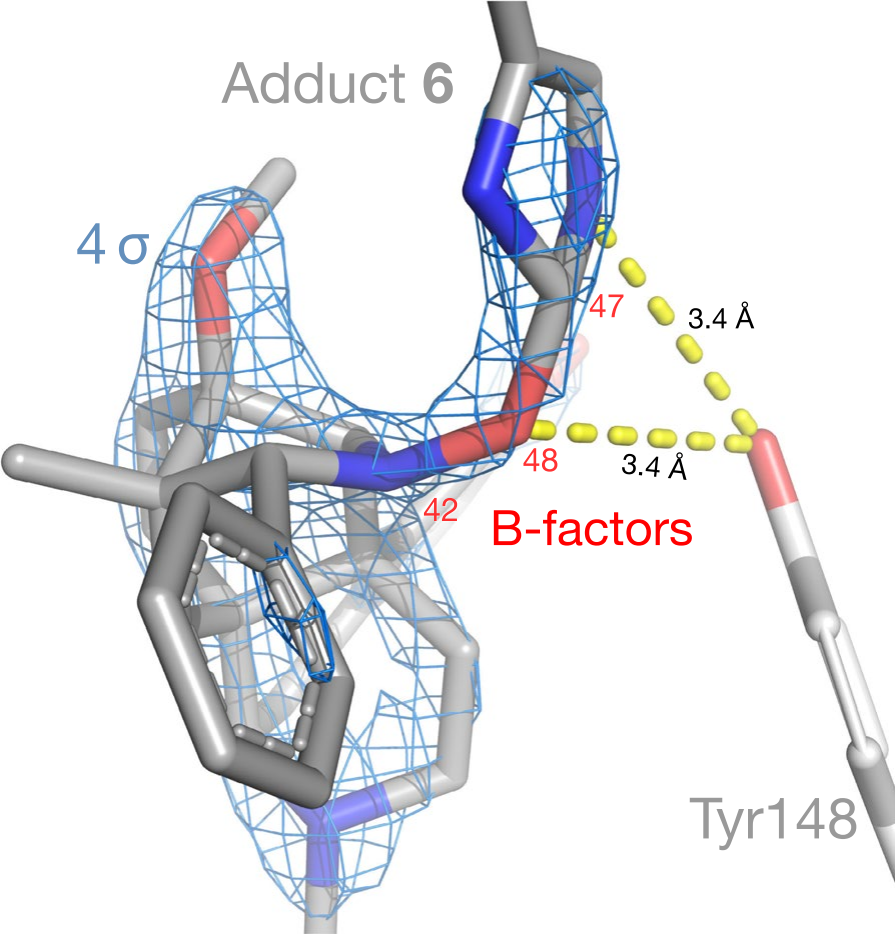
Fit of adduct **6** to density, with B-factors (*red*) and polar contacts to Tyr148^3x33^

Substituting adduct **6** for His54 and BU72 gave an excellent fit, with no excess or unexplained density even at 2 σ (Fig. 5; data in Additional files 3, 4, 5 and 6) [41]. Both bonds to oxygen were of typical length (1.5 Å) and were resolved up to 4.2 σ—that is, higher density than most of the ligand itself and surrounding side-chains. Unlike Mg^2+^, the oxygen atom was well centered in the density. Oxygen also gave a superior B-factor to Mg^2+^, both lower and more consistent with its bonding partners, making this a much more plausible candidate element (Fig. 5) [15]. The lower B-factor for oxygen results in a more precise fit (DPI 0.14 vs 0.22 Å). Indeed, it is among the most precisely-resolved atoms in the entire structure. The bridging oxygen and modified histidine moiety make favorable polar contacts with Tyr148^3x33^, which are close to the length of weak hydrogen bonds.

### The adduct is highly strained

The bound geometry of adduct **6** gave acceptable ligand validation metrics, which were superior to the original model of BU72, **1a** (Table 1; data in Additional file 7).

**Table 1:** Ligand validation: geometry relative to Grade restraints, and electron density fit from PDB validation reports

PDB structure	5C1M (v1.5) [8]	8E0G [41]
Ligand	BU72 (**1a**)	adduct **6**
*Geometry*		
Geometric outliers (|*Z*|> 2)	26	10
Severe outliers (|*Z*|> 5)	9	1
Bond angle root mean square *Z* (RMSZ)	3.23	1.52
Bond length root mean square *Z* (RMSZ)	3.32	1.13
*Fit to electron density*		
Real-space correlation coefficient (RSCC)^a^	0.914	0.951
Real-space R (RSR)	0.090	0.081

^a^Lower values are better except for RSCC

**Fig. 6:**
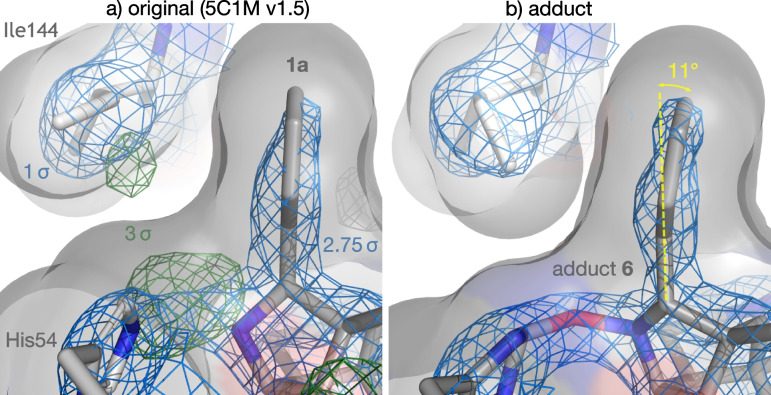
Fit of phenyl group to adjacent residue Ile144^3x29^, shown with solvent-accessible surfaces. **a** Original model (5C1M v.1.5). **b** Adduct

The only severe outlier was the bond angle at the bridging oxygen (131° vs the ideal 109°: *Z* = 7.2). There are several indications that this is real strain rather than a fitting artifact, however. The angle is clearly resolved at high density and is consistent with tension from the tethered N-terminus. The phenyl group is bent 11° out of plane, consistent with being pulled against the adjacent residue Ile144^3x29^ by the same tension (Fig. 6b). This bend is also clearly resolved and is comparable to those seen in severely strained aromatic residues at subatomic resolution [42]. It also yields a more complementary fit to Ile144^3x29^ than the original model, as well as eliminating another small pocket of unexplained density (Fig. 6).

**Fig. 7:**
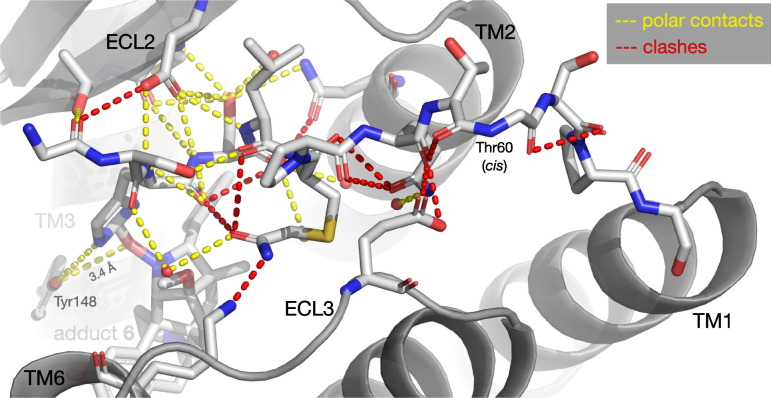
Polar contacts (< 3.6 Å) and clashes of the N-terminus in the adduct model. Note the high-energy *cis*-peptide bond at Thr60

Strain is also evident in the N-terminus itself: in both this model and the original (5C1M v.1.5), Thr60 adopts a rare and high-energy *cis*-peptide bond, and there are many energetically unfavorable clashes along the peptide backbone (Fig. 7).

**Fig. 8:**
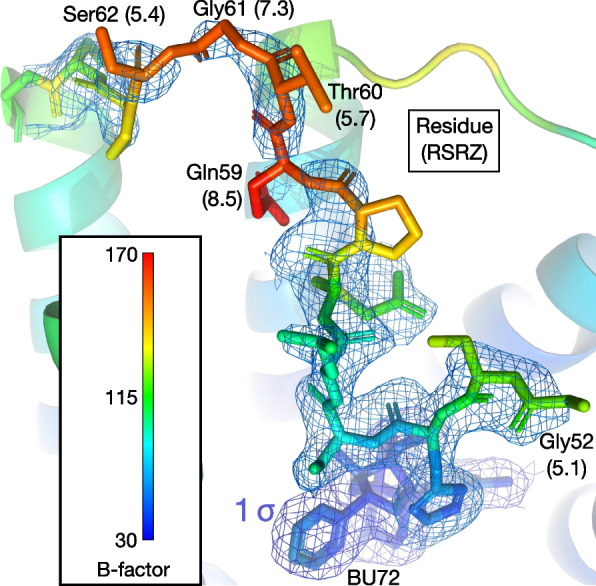
The N-terminus in the revised original model (5C1M v.2), colored by B-factor. Note poor electron density coverage for some residues; severe RSRZ outliers (> 5) are given in brackets

Alternate modelling can eliminate the *cis*-peptide bond, as in the revised version of the original model (5C1M v.2). However, this results in a worse fit to the density, which is extremely weak in this region: several side-chains and even parts of the backbone are unresolved at 1 σ, yielding eight RSR outliers in the N-terminus, five of which are severe (Fig. 8). Atomic displacements in the N-terminus are also extremely high: the occupancy-weighted average B-factor (OWAB) of the last seven residues (58–64) are higher than 95% of residues in the structure. Indeed, Gln59 has the highest value in the entire structure, 159 Å^2^, compared to a median of 46. The above features (poor density coverage, high B-factors, clashes and a probable *cis*-peptide bond) establish that the N-terminus is constrained in an extremely unfavorable high-energy state by the tethered ligand.

## Discussion

### The adduct and nanobody influence the receptor, confounding inferences about the G protein-bound active conformation

The adduct may lead to mistaken inferences about the full-length receptor. For instance, some have interpreted the N-terminal “lid” as part of the binding pocket, consistent with the contraction of that pocket commonly observed in active G protein-coupled receptors (GPCRs) [43]; however, the many later active μOR structures all lack this feature (Table 2). Others have interpreted the lid as evidence that agonist binding stabilizes the N-terminus [44], but as noted above, the N-terminus is in fact extremely strained and unstable.

**Table 2:** All PDB structures to date containing active μOR or Nb39, with inactive μOR for comparison

PDB	Species		Bound to		Ligand	Resolution	Source
	**Mouse**	**Human**	**Nb39**	**G**_**i**_		(Å)	
*Active μOR*							
5C1M	•		•		BU72	2.1	[3]
6DDF	•			•	DAMGO	3.5	[45]
7SBF	•			•	PZM21	2.9	[46]
7SCG	•			•	FH210	3.0	[46]
7T2G	•			•	Mitragynine pseudoindoxyl	2.5	[47]
7T2H	•			•	Lofentanil	3.2	[47]
7U2K	•			•	C6 guano	3.3	[48]
7U2L	•			•	C5 guano	3.2	[48]
8EF5		•		•	Fentanyl	3.3	[49]
8EF6		•		•	Morphine	3.2	[49]
8EFB		•		•	Oliceridine	3.2	[49]
8EFL		•		•	SR-17018	3.2	[49]
8EFO		•		•	PZM21	2.8	[49]
8EFQ		•		•	DAMGO	3.3	[49]
8F7Q		•		•	β-endorphin	3.2	[50]
8F7R		•		•	Endomorphin-1	3.3	[50]
*Active κOR*							
6B73	•		•		MP1104	3.1	[51]
7YIT		•	•		Nalfurafine	3.3	[52]
*Inactive μOR*							
7UL4	•				Alvimopan	2.8	[53]

Species, binding partner and resolution are indicated. PDB entry 6DDE is omitted: 6DDF is a higher-resolution analysis of the same dataset

Another factor influencing the receptor conformation is the intracellular binding partner used, the G protein-mimetic nanobody Nb39. This is evident in the largest movement during activation, involving TM6. Viewed from the intracellular end, TM6 pivots outwards and rotates clockwise; this “macroswitch” occurs during activation of all GPCRs studied to date [54, 55]. Different coupling partners would be expected to promote different shifts, and indeed nanobodies yield different receptor conformations than G proteins [54].

**Fig. 9:**
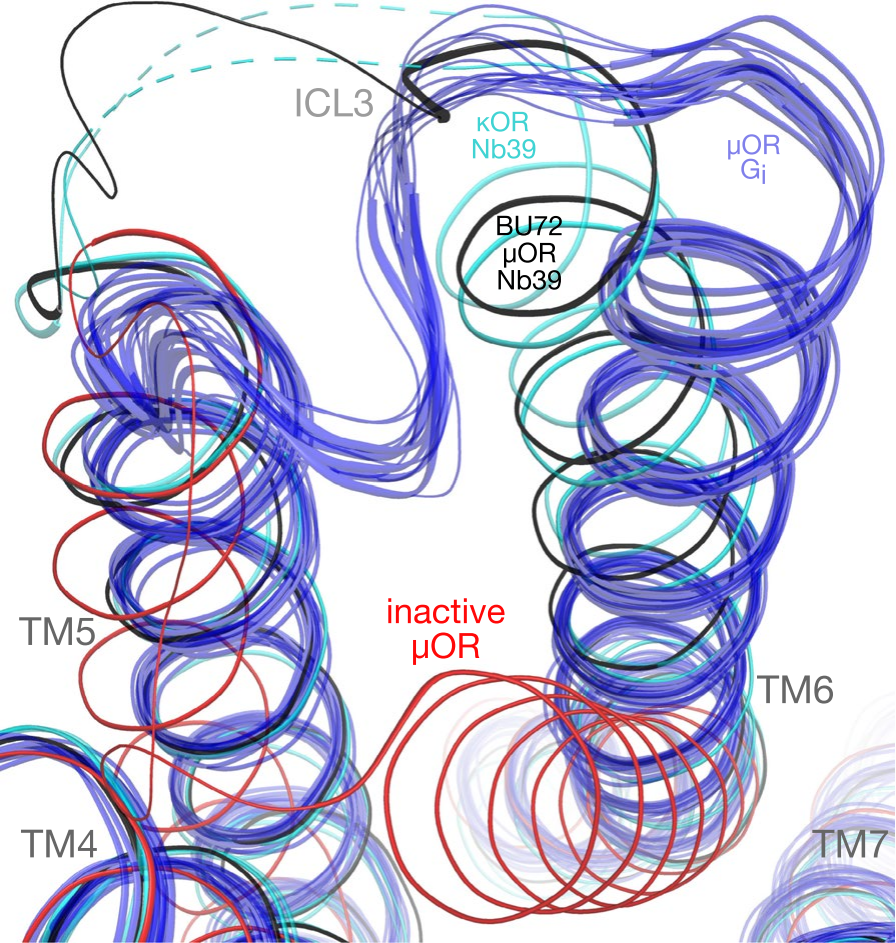
Overlay of opioid receptor structures showing TM5, TM6, and ICL3 (inactive, Nb39-bound, and G_i_-bound). The full-length receptors were aligned against DAMGO-bound human μOR. See Table 2 for PDB identifiers and other details

The first structure of active μOR bound to G_i_ protein differed markedly from the BU72-bound structure around TM6, which was tentatively attributed to the effect of nanobody Nb39 [45]. The many subsequent G_i_-bound μOR structures support this proposal. All G_i_-bound structures cluster very tightly in this key region (Fig. 9), despite featuring diverse μ opioids bound to μOR from different species (Table 2). The BU72-bound structure is a clear outlier, with TM6 much closer to TM5, and rotated in the opposite direction. As a result, intracellular loop 3 (ICL3) bunches outwards in a disordered loop, rather than being pulled into a helix as in the G_i_-bound structures. Strong confirmation that these differences are caused by the nanobody come from two later structures of the active Nb39-bound κ opioid receptor (κOR), which show the same discrepancies (Fig. 9).

**Fig. 10:**
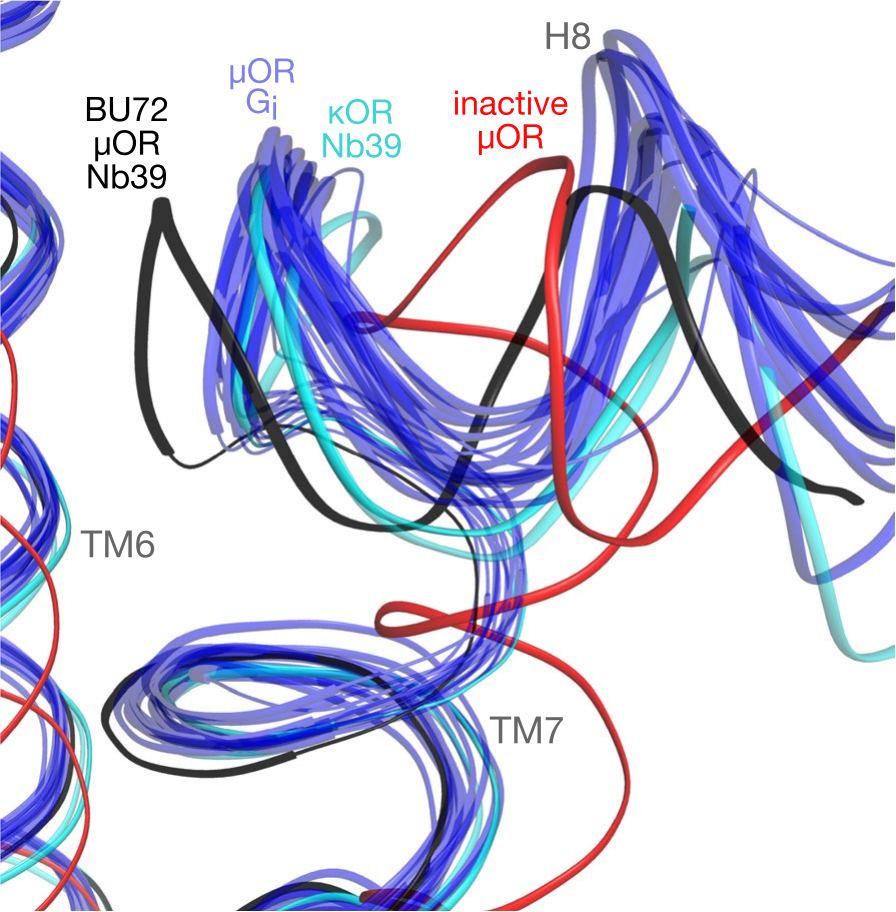
Overlay of H8 in opioid structures (inactive, Nb39-bound, and G_i_-bound). See Table 2 for PDB identifiers and other details

Another discrepancy between the BU72-bound structure and the others is in helix 8 (H8). Activation of class A GPCRs, such as opioid receptors, involves an inward shift of H8, making and breaking contacts at its base [55]. Relative to the inactive state, the base of H8 shifts noticeably more in the BU72-μOR structure than in the others, which all cluster tightly (Fig. 10). The consistency of the other structures, both G_i_- and Nb39-bound, suggests that the nanobody is not responsible for this discrepancy. Likewise, the great diversity of peptide and small-molecule ligands used (Table 2) suggests that ligands also have little effect on H8. Rather, some other factor such as adduct-induced distortion appears to be responsible. Some distortion is to be expected, since the forces restraining the ligand and N-terminus in high-energy conformations must act equally on the rest of the receptor. Compounding this, the N-terminus is involved in numerous polar contacts and clashes with surrounding residues (Fig. 7), and these forces must also influence the receptor conformation.

Thus, the BU72-bound structure is a clear outlier from the many later G_i_-bound structures in features associated with receptor activation. In addition to the nanobody, it appears that the adduct contributes to these differences. The consistency between the G_i_-bound structures establishes them as superior templates for modelling the active G protein-bound state of the receptor, despite being of lower resolution (Table 2).

The μOR binds to other proteins, such as arrestins and kinases, and the Nb-39-bound state may be of interest in modelling alternative active states. However, while there is ongoing debate about whether some adverse effects of μ opioids are arrestin-mediated, there is a strong consensus that analgesia is G protein-dependent [56]. Thus, this is the effector of most interest in opioid development.

### Possible methods for adduct structure determination

In the original study, a search for alternative ligands to account for the unexplained density was unsuccessful. The mass spectrum of the crystallization mixture revealed a molecular ion consistent with BU72, but no others of similar mass [3]. However, the intact adduct would not be detectable in solution. If it decomposed, one decomposition product per binding site would yield negligible concentrations relative to saturating BU72. An alternative test would be for modification of His54: proteolysis of the receptor and mass spectrometry of the fragments should reveal either the adduct or decomposition products. A simpler alternative would be to substitute a short H-Gly-Xaa-His-containing peptide for the receptor, although this might also result in side-reactions. The initial nickel complex itself should be detectable spectroscopically and may indeed give a noticeable yellow color to the solution [24].

An obstacle to isolation of the adduct may be instability. Previously reported adducts **4** and **5** were not isolated, but detected only by mass spectrometry as reaction intermediates [21, 40]. However, the tethered conformation of the N-terminus separates Gly52 from His54, rendering a nickel complex between the two residues impossible (Fig. 8). Thus, adduct formation would liberate the ion and break the catalytic cycle. Moreover, the N-terminus almost entirely occludes the binding pocket, leaving only a narrow tunnel to the ligand [3]. Thus, the adduct bonds are sterically shielded, which may inhibit further reactions.

### The risk of similar reactions elsewhere, and precautions against ROS generation

The risk of unexpected complexes and oxidations like this is not specific to the structures discussed here. The conditions that led to these reactions, in both this case and previously [25], are widely used. Many proteases commonly used for the cleavage of fusion proteins leave glycine as the N-terminal residue (e.g., thrombin, factor Xa, tobacco etch virus protease, and rhinovirus 3C protease) [57]. Unsurprisingly then, the N-terminal H-Gly-Xaa-His- motif is common in the Protein Data Bank, appearing in > 7000 sequences (~ 4% of the total). Nickel affinity columns are also widely used. Many of these proteins would therefore be expected to form Ni^2+^-Gly-Xaa-His- complexes. However, the first residues of the N-terminus are almost invariably disordered: 97% of human proteins have disordered terminal residues [58], and 42% of all disordered residues are in the N-terminus [59]. Thus, these complexes are unlikely to be resolved and are therefore likely to go undetected. Peroxide-generating buffers such as HEPES are also ubiquitous; thus, quite common procedures for protein preparation inadvertently generate ROS. Oxidation by ROS can have many undesirable effects on proteins, from modifying side-chains (which may influence the overall conformation) to cleaving the amide backbone [60].

The possibility of reactions like this should be considered in the choice of truncation sites and purification conditions for protein isolation. Generation of nickel complexes, ROS, and subsequent reactions could be prevented by choosing a different cleavage site (with a third residue other than histidine) or a nickel-free purification method. Where a nickel complex is desired, for instance to promote crystallization [25] or assist in phasing [27], a non-piperazine buffer could be used to avoid or reduce ROS generation [61].

## Conclusions

In summary, the density observed between BU72 and His54 is not consistent with non-covalent interactions or a metal coordination complex, and must instead represent two covalent bonds to a non-metal atom, approximately isoelectronic with Mg^2+^. While this evidence does not unambiguously identify the bridging atom, it is inconsistent with the original model of ligand and receptor as distinct molecules. The use of conditions known to generate ROS, along with previous reports of adduct formation in the presence of ROS, suggests the possibility of an oxygen-bridged adduct here. All features examined are consistent with this proposal.

The structure differs in key respects from subsequent structures of μOR bound to G_i_ protein, partly due to the use of a nanobody; however, strain within the tethered N-terminus, and its contacts with surrounding residues, also appear to contribute. These subsequent structures are likely to be more accurate templates of the active, G protein-bound receptor for ligand docking and receptor modelling. Oxidative artifacts like this could be prevented by using different truncation sites or purification conditions.

## Methods

Starting from the previously reported model of μOR with **1b** [5], Mg^2+^ was added to the center of the unexplained density with sphere refinement using Coot [62] in CCP4i2 [63], and uploaded with the original structure factors to PDB-REDO server [64] for automated refinement. The resulting complex was submitted to CheckMyMetal [14] for validation; all suggested alternative metals were also submitted.

The ideal structure and geometric restraints of the **1b**-histidine adduct **6** were generated using Grade server [65]. BU72 was deleted from the original model, His54 was mutated to the adduct, and the model fitted and refined as above. Because the PDB validation report did not evaluate the geometry of adduct **6**, ligand distortions in the bound ligands were tabulated in Coot and used to calculate *Z* scores, comparing ideal values and standard deviations from Grade with modelled values for **1a**, **1b**, and **6** (Additional file 7). Diffraction precision indexes were calculated using Online DPI [12]. Protein structures were aligned and visualized using Pymol [66]. Figures were annotated using Inkscape [67]. The interactive comparison of the original and adduct models was created using Molstack [68]. Structural formulae were drawn using Marvinsketch [69] and are provided in Chemical Markup Language as Additional file 8.

### Supplementary Information

**Additional file 1.** Coordinates of the BU72-Mg^2+^-µOR complex (mmCif).

**Additional file 2.** Structure factors of the BU72-Mg^2+^-µOR complex (MTZ).

**Additional file 3.** Coordinates of the BU72-µOR adduct (mmCif).

**Additional file 4.** Structure factors of the BU72-µOR adduct (MTZ).

**Additional file 5.** Ideal coordinates of BU72-histidine adduct **6** from Grade server (PDB).

**Additional file 6.** Geometric restraints of BU72-histidine adduct **6** from Grade server (Cif).

**Additional file 7.** Geometric outliers of BU72 and the BU72-µOR adduct calculated from Grade server values (xlsx).

**Additional file 8.** Chemical structures (structural formulae) of the small molecules (CML).

## Data Availability

Coordinates and structure factors for the adduct have been deposited in the Protein Data Bank (accession number 8E0G [41]). These and other datasets supporting the conclusions of this article are included in the Supplementary Information. Earlier versions of this work were published on a preprint server [70]. An interactive comparison of the adduct and original model, including electron density, is available at: https://molstack.bioreproducibility.org/p/Y7FU.

## Abbreviations

DAMGO: [D-Ala^2^, N-MePhe^4^, Gly-ol]-enkephalin
DPI: Diffraction precision index
GPCR: G protein-coupled receptor
H8: Helix 8
HEPES: 2-[4-(2-Hydroxyethyl)-1-piperazinyl]ethanesulfonic acid
ICL: Intracellular loop
OR: Opioid receptor
OWAB: Occupancy-weighted average B-factor
PDB: Protein Data Bank
PIPES: 1,4-Piperazinediethanesulfonic acid
RMSZ: Root mean square *Z* score
ROS: Reactive oxygen species
RSCC: Real-space correlation coefficient
RSR: Real-space R value
s.d.: Standard deviation
TM: Transmembrane helix
UNX: Unidentified atom
Xaa: Any amino acid
